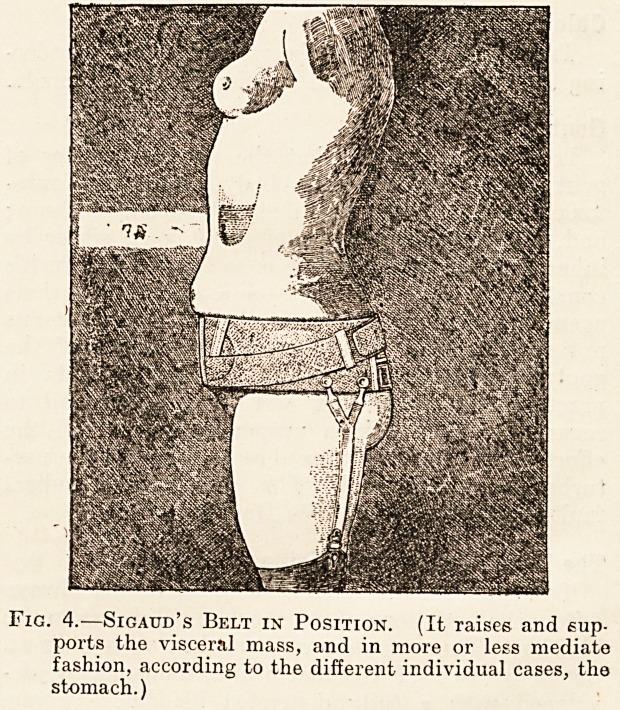# The Girdle-Test (l'Epreuve de la Sangle)

**Published:** 1910-11-05

**Authors:** A. Chaillou, Leon MacAuliffe

**Affiliations:** de l'Institut Pasteur; Chief Editor of "La Clinique," Paris.


### Hospital Clinics.

### THE GIRDLE-TEST (L'EPREUVE DE LA SANGLE).

By A. CHAILLOU, M.D. (de l'Institut Pasteur) and LEON MacAULIFFE, M.D., Chief Editor of "La Clinique," Paris.

In the course of his work on prolapse Glénard has several times described a clinical indication which, although many doctors do not yet know of it, is of considerable moment; we are speaking of the girdle-test (*l'épreuve de la sangle*).[1] It was not, however, until C. Sigaud and Léon Vincent, of Lyons, published their valuable treatises[2] that the

Fig. 1.—First Movement of the Girdle-test. (Side view. The doctor's hands are raising the abdominal mass, but without crushing it.)

full value of this indication was established. These two authors gave the exact interpretation of the phenomenon described by Glénard and completed his researches into this matter.

### Technique.

The patient stands, and the doctor, standing behind him, makes both hands meet in front of the subject's abdomen, just above the pubis, so as to form a regular girdle; the abdominal mass is thus raised, and at the same time the viscera are more or less pressed back. This pressing back of the viscera is gently increased until the patient experiences relief and a sensation of support and comfort. Sometimes one is obliged to penetrate deep into the abdomen to produce this sensation and to press in the wall above the pubis hard, just as would a big pad. This operation constitutes the first movement (*le premier temps*) of the girdle-test. It is continued until the most favourable degree of pressure, that which affords the greatest amount of relief to the patient, has been ascertained (fig. 1).

By a second movement (*deuxième temps*) (fig. 2) the doctor removes his hands abruptly and allows the whole abdominal mass to fall together. The pain thus excited varies; sometimes it is so intense as to cause giddiness and even syncope. Patients behave very differently when examined for this clinical indication.

The girdle-test is called *negative* when both movements leave the patient unaffected; neither the one nor the other gives him relief or discomfort. Patients belonging to this numerous category are generally suffering from merely slight maladies.

The *complete positive* test produces a sensation of comfort in the first movement and of more or less acute pain in the second. The test is also called *positive* if it produces either a sensation of comfort in the first movement or one of pain in the second movement. In the first case the test is *positive in the first movement*, in the second case it is *positive in the second movement*. In both of these varieties one of the movements leaves the patient unaffected; it produces no sensation.

We have said that the degree of raising which

Fig. 2.—Second Movement of the Girdle-test. (The doctor removes his hands abruptly and allows the visceral mass to fall.)

is able to produce a sensation of relief varies extremely. Sometimes mere contact and scarcely any pressure above the pubis imparts a feeling of comfort. The test is then called *simply positive*. At other times strong pressure must be applied above the pubis, and with such exertion that the wall of the abdomen projects in a roll above the doctor's hands and falls over them; the test is then called positive with the pad (positive *avec pelote*).

There is a very limited category of patients to whom the hands, far from giving relief as they carry out the first movement of the girdle-test, cause some pain and even oppression: these patients regain comfort only in the second movement, when the doctor abruptly stops supporting the abdomen. The test is then called *paradoxical*. It does not, like the negative test, leave the patient unaffected; it causes a feeling of pain in the first movement, and the feeling is carried to such a pitch that this first operation could not be continued for long.

### The Significance of the Girdle Test.

The girdle-test is only to be understood by studying the evolution of the digestive tract. In the first part of the patient's life the digestive tract hypertrophies and dilates at the same time as the abdominal wall covering it. This process makes its appearance generally at the end of the period of growth, when the subject grows stouter and his muscles reach their maximum size by becoming slightly permeated with fat. The digestive tract does not only hypertrophy and dilate throughout its parts, but it also drags upon and, therefore, lengthens all its peritoneal coils and all its supporting ligaments. Being, as it were, too much straitened within the abdominal cavity in spite of the concomitant stretching of the walls, it drives the solid organs, and especially the liver, outwards to the periphery. The abdominal walls undergo continuous outward pressure. The posterior wall, formed by the spine and sacro-lumbar mass, resists and does not experience deformation.

The concavity of the upper wall, formed by the diaphragm, becomes more marked, and this to such an extent that the lungs may be pushed back and hindered in their function. This partly accounts for the shortness of breath noticeable in the beginnings of obesity. The lower wall, formed by the base of the pelvis (*plancher pelvien*), tends to become more hollowed out, and the pelvic organs drag upon and tend to lengthen their ligaments. In a woman this is the period of uterine version or flexion, with or without cystocele and rectocele.

But it is, above all, the entirely musculo-aponeurotic, antero-lateral wall which is affected by this pressure from within, hence it must give way sooner or later, according to the quality of the muscular tissue.

There follows upon this phase of hypertrophy and of dilatation of the digestive apparatus a second phase of atrophy and retraction.[3] Atrophy and retraction do not take place in completely parallel fashion; according to the nature of the tissues one or the other predominates, and this predominance it is which regulates the variations in shape of the abdomen during this period of decline, just as hypertrophy had regulated them during the first period.

The walls of the digestive organs grow thinner, but the calibre itself of the hollow digestive viscera becomes similarly reduced. In extreme cases the colon, becoming smaller and smaller, is reduced to the thickness of the little finger. The atrophied descending colon is pushed inwards towards the spine. The cæcum, which was at first the shape of a big ampulla, dwindles into a little hard and wrinkled pudding. The stomach atrophies; its large curve, which had been near the navel, is drawn up towards the false ribs.

The mass of the small intestine, which used to form a taut band, a regular elastic cushion in the hypogastrium, gives no sign of existence when the patient is lying on his back; it has disappeared into the depths of the abdomen, and cannot be traced by the fingers engaged in deep palpation.

### Some Anatomical Modifications.

On the other hand, the liver is no longer supported or pushed back into the concavity of the diaphragm; it drops and appears below the false ribs, at first only during inhalation, then gradually it ceases from rising and becomes accessible to the touch for a great portion of its extent.

Quite different has been the action of the suspensory ligaments of all these organs. Being formed of connective tissue and unstriped fibre, they had suffered distension and, during the phase of hypertrophy, they had become permeated with fat, which had dissociated their elements. Then, when the phase of atrophy followed, this fat disappeared; the dissociated connective fibres remained lengthened; the atrophied and degenerate unstriped fibres are unable to effect any appreciable retraction, and the result is that the mesos and ligaments, which have grown too long, can only feebly sustain viscera, which have become too small.

A nearly identical mechanical process has brought about deformation of the antero-lateral abdominal wall. At the outset, the muscles have hypertrophied under the action of pressure from within. In spite of the crossed fibres of the obliqui, the transversi, and the recti, an arrangement which would seem to secure remarkable cohesion of structure for this wall, the fibres have suffered distension and dissociation. The aponeurotic cells, too, have grown bigger.

When the period of atrophy supervenes, these cells, the walls of which may have become thinner, remain too big; the slightly degenerate muscular fibres no longer retract completely, but remain lengthened. And the wall, as a whole, having been stretched, has lost elasticity; it forms too flaccid and weakened a veil, stretching from the false ribs to the pelvis, and no longer supporting the entire mass of the abdominal viscera. Henceforward the viscera will be exposed to continual shaking; at the patient's slightest movement they will be much displaced since the displacement cannot be properly restricted.

One can easily imagine how the varying phases of the evolution of the digestive tract would react upon the organs and create for them quite different physiological conditions, since atrophy necessarily entails mobility and shaking of the viscera.

By strengthening the walls, by diminishing the abdominal cavity, and limiting the movements of the intestines and of the organs, the girdle-test at once alters the conditions under which these are fulfilling their functions, and gives rise to sensations which vary according to the patients.

These notions of pathological physiology were necessary in order to understand and interpret all the functional indications observed in the course of examination by the girdle-test.

### Significance of the Girdle-test.

The girdle-test is positive generally at an early period of the individual's evolution. The mobility of the viscera and the flabbiness of the wall are excessive; but more or less vigorous pressure will counteract the defectiveness of the wall and keep the organs sufficiently steady to bring about an immediate sensation of relief.

Many of those patients whose test gives a very distinct positive result will see a complete, or nearly complete, end put to their troubles by wearing an appropriate belt. This secures to them permanently those fresh conditions of abdominal tension which were afforded temporarily by the doctor's hands, when they raised and supported the mass of the viscera. A marvellous therapeutic result is thus obtained especially with muscular patients in the period of decline, whose physical existence

Fig. 3.—Sigaud's Belt. (The dotted line shows the position of the pad in the middle, which is supposed to be seen through the thickness of the material.)

already places too great a strain upon their muscles. The habitual wearing of a belt allows them to endure cheerfully fatigues which had been prohibited for a long time.

### The Development of the Displacement.

However, although prolapse derives from relaxation of the muscles and ligaments, it must not be thought that a belt is necessary or even of use in all cases. "The belt meets the case of a fact of sensibility and not of visceral displacement" (Sigaud). Now, certain individuals possess but little organic consciousness (to use an expression which has been much in vogue for some time); they feel but little, and the doctor's help, as he applies the girdle-test, is needed to enable them to recognise and combat this increased sensibility. Other very sensitive patients, on the contrary, apply an abdominal belt, generally made of flannel or crêpe, of their own accord before consulting a doctor. In the immense majority of cases, as the relaxation of muscles and ligaments becomes more pronounced, abdominal hyperæsthesia increases; the jolting and the displacement of the viscera are endured with difficulty, and the application of a good belt suffices to relieve the patient when the ill-health is of recent development.

On the other hand, it is a physiological law that anæsthesia or hypoæsthesia follows upon hyperæsthesia. And so one need not be astonished by the apparently paradoxical fact that it is generally the oldest cases of prolapse which show the least sensitiveness in the girdle-test, and which derive the least benefit from wearing a belt. Hence the paradoxical test is found in patients who have reached a more advanced stage of illness.

### Sigaud's Belt.

As this evolution progresses, the viscera, which are pulled and shaken about in an abdominal cavity too big for them, end by no longer reacting painfully in response to this shaking and pulling: reactivity becomes so blunted that their very incomplete and irregular functions can be carried out only under the continual action of the displacements which they undergo and which might be called traumatic. Let these viscera be supported

Fig. 4.—Sigaud's Belt in Position. (It raises and supports the visceral mass, and in more or less mediate fashion, according to the different individual cases, the stomach.)

and kept steady by any means whatever; let the violent causes which perpetually stimulate them suddenly cease, and they will be upset in their functions and react painfully. Indeed, a belt cannot be tolerated by such a patient; it increases his discomfort or else he breaks down under it completely.

For this category of patient there is but one treatment: absolute repose, often prolonged for a great interval of time, in bed. At first lying on his back increases the patient's discomfort, but after a varying amount of time (a fortnight, a month, or more) he pulls himself together, and the digestive tract gradually begins to work again at rest. Then, but not till then, a belt will bring about a cessation of pain; it will be worn with comfort, and will enable the patient to resume a more or less active life.

The belt that we and many of our comrades have used now for seven years is Sigaud's belt (fig. 3), which all bandage-makers manufacture on the pattern here given.

It is of the same shape as the belt that Glénard formerly used, made of strong elastic material (*soie damier*), not very deep (about 2¼ inches to 4 inches), and, if the occasion arises, it has in the middle of it an elastic pad which, fifteen years ago, was called by its maker the hypogastric pad.

Moreover, this belt is supplied with an additional band and two suspenders. It is worn as follows: The middle and lower portion of the pad is placed just above the pubic symphysis. The belt itself, which must be fixed quite horizontally, passes round the sides below the anterior iliac spines and fastens very low behind at the level of the sacral region. In this position the additional band prevents the lower edge of the belt from projecting in front. Fig. 4 shows the proper position of the belt.[4]

[1] Glénard: "Des ptoses." *Rapport à la Soc. de Méd. de Paris* (Paris: Alcan).

[2] C. Sigaud: "Traité Clinique de la Digestion" (Paris: Doin, t. I. 1900, et t. II. 1908). Leon Vincent: "Traité de l'Exploration Manuelle des Organes Digestifs" (Paris: Doin, 1898). Nachmann: "Tension Abdominale et Palper de l'Abdomen d'après la Méthode de Sigaud, de Lyon" (Thèse de Paris, 1908).

[3] Chaillou et MacAuliffe: "Précis d'Exploration Externe du Tube Digestif" (Paris: Maloine, 1903). See also *Lancet*, February 5, 1910, pp. 361-364.

[4] One of us has verified with the radioscope the manner in which the viscera are held together by the action of this belt, and what he saw confirms all the details given in this article.

## Figures and Tables

**Fig. 1. f1:**
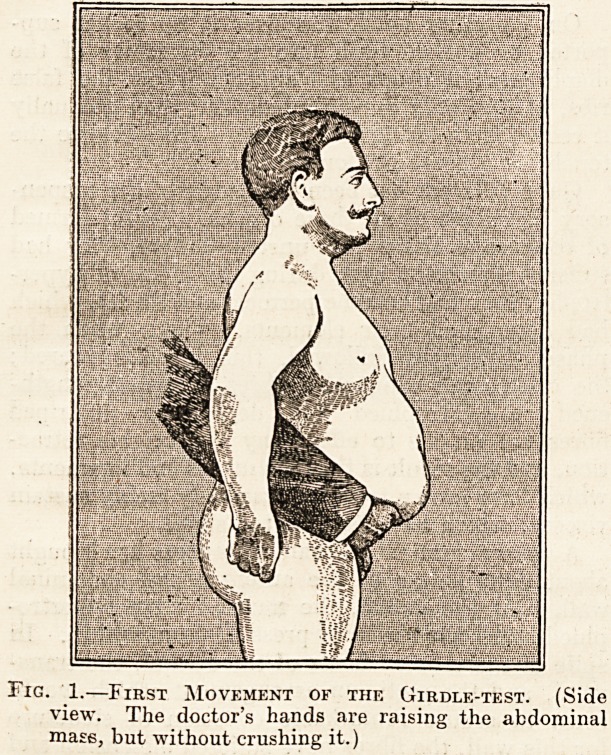


**Fig. 2. f2:**
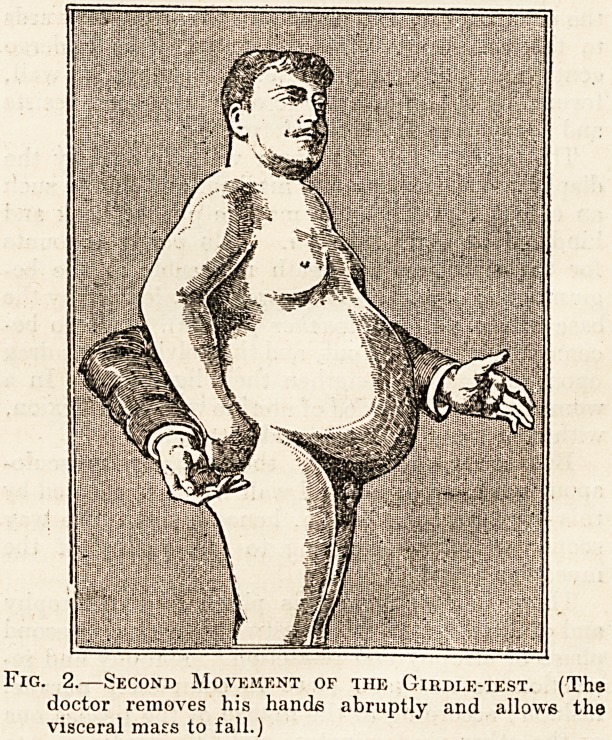


**Fig. 3. f3:**
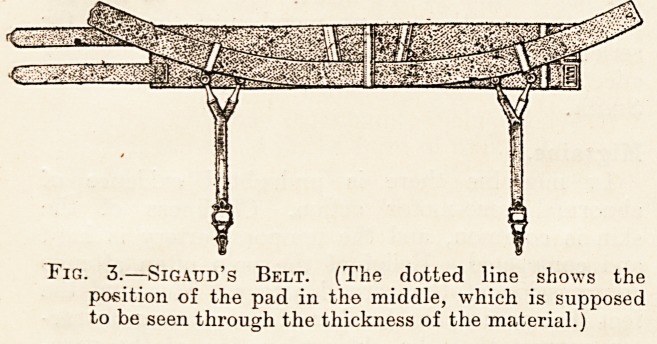


**Fig. 4. f4:**